# Supplementary material for: Multi-model machine learning for automated identification of rice diseases using leaf image data
Source: PLoS One. 2025 Sep 16;20(9):e0307461. doi: 10.1371/journal.pone.0307461 (PMC12440219; doi:10.1371/journal.pone.0307461)
Supplement: Attachment — Submitted filename: RTR_for_Rice leaf.docx [file pone.0307461.s001.docx]

**RESPONSES TO REVIEWERS’ COMMENTS AND SUGGESTIONS**

**Journal: PLOS ONE
Manuscript Number: PONE-D-24-27739  
Article Type: Full-Length Article
Title: Multi-Model Machine Learning for Automated Identification of Rice Diseases Using Leaf Image Data**

We thank reviewers, academic editor, and the editor for their time, constructive comments, and useful suggestions. We have addressed the comments of the reviewers to the best of our ability. Our responses to the reviewers’ comments (bold letter) are in blue and in red are the addition, deletion, or modification in the revised manuscript.

**Reviewer #1:**

**Comment#1: Adapt the abstract highlighting motivation, contribution and innovation.**

Response: Thank you for your suggestion to enhance the abstract by better highlighting the motivation, contribution, and innovation of the study. Based on your feedback, we have revised the abstract.

Purpose: Rice, a staple meal for about half of the world's population, is critical to global food security, especially in Asia. However, diseases have a severe impact on rice production, resulting in significant yield losses or outright crop failure. Traditional techniques of identifying rice diseases are time-consuming, labor-intensive, and rely heavily on specialist knowledge. As a result, a rapid, cost-effective, and automated method for detecting rice illnesses is critical for modernizing agricultural techniques and ensuring sustainable food production.

Methods: This paper presents a novel hybrid deep-learning and machine-learning framework for automatically identifying rice plant diseases from leaf photos. The MobileNetV2 architecture is used for efficient feature extraction, which yields deep, representative features from input photos. These features are then classified using machine learning classifiers with various kernel functions, which apply a strong 10-fold cross-validation technique to assure model reliability.

Results: Using a medium neural network, the suggested system achieves world-class performance, with a classification accuracy of 98.6%, specificity of 98.85%, and sensitivity of 97.25%. The framework is computationally efficient and scalable, allowing for greater dataset testing.

Conclusions: The invented technique provides a dependable and efficient solution for the early diagnosis of rice illnesses, minimizing farmers' reliance on manual knowledge and allowing for timely intervention. Future study will investigate how to integrate the system with cloud-based platforms and IoT-enabled mobile applications for real-time, remote monitoring in disconnected agricultural settings.

**Comment#2: What is medium neural network? is this standard terminology to refer NN?**

Response: Thank you for pointing this out. The term "medium neural network" was used in the manuscript to describe the neural network architecture with a moderate number of layers and parameters, designed to balance computational efficiency and classification performance.

**Comment#3: is the method applicable other than India? Hunger is a global problem.**

Response: Thank you for raising this important point. While the dataset used in our study is specific to rice plant diseases commonly found in India, the proposed method is not limited to this region. The hybrid deep-learning and machine-learning framework we developed is generalizable and can be adapted to detect rice diseases in other geographical regions, provided that region-specific datasets are used for training and validation.

**Comment#4. Introduction is unnecessarily long, make it preces and clear.**

Response: Thank you for your valuable feedback. We agree that the introduction can be made more concise and focused. In response to your comment, we have revised the introduction to streamline its content, ensuring it clearly communicates the motivation, problem statement, objectives, and significance of the study without unnecessary elaboration.

**Comment#5. Literature Review section can be added and more literature can be discussed on crop disease detection using recent technology. For example**

**a) Recent advances on crop disease detection using UAV**

**b) Tomato Leaf Diseases identification Using an Explanation-Driven Deep-Learning Model**

**c) Machine learning methods for precision agriculture with UAV imagery**

Response: Thank you for your insightful comment and for suggesting additional areas to strengthen the manuscript. We agree that incorporating a dedicated literature review section would provide a more comprehensive context for our study. In response, we have added a new "Literature Review" section to the manuscript, which discusses recent advancements in crop disease detection using modern technologies, including the references you mentioned.

**Comment#6. Data labelling procedure is not clearly mentioned. are there any plant pathologist involved in this process? otherwise, how do you validate the ground truth?**

Response: Thank you for raising this important concern. We understand the significance of clearly describing the data labeling procedure and ensuring the accuracy of ground truth in disease detection studies. In response, we have clarified the data labeling process in the revised manuscript as follows:

Data Labeling Procedure: The dataset for this investigation consists of tagged images acquired from publically available sources and agricultural research sites. Each image was pre-labeled with the appropriate disease class based on the metadata provided by the data sources. To guarantee consistency, we carefully checked the labels to assure their accuracy.

**Comment#7. I assume CNN can extract feature automatically, why author are using feature extraction as separate step?**

Response: Thank you for raising this question. You are correct that convolutional neural networks (CNNs) are capable of automatically learning and extracting features from raw image data during the training process. In future work, we plan to explore end-to-end CNN models and compare their performance with the hybrid approach to assess trade-offs in terms of computational complexity and classification accuracy.

Based on the reviewer suggestion, we have removed the separate subsection from the revised manuscript.

**Comment#8. Why author are discussing YOLO in methodology for object detection as the proposed task is classification?**

Response: Thank you for your comment and for pointing out the apparent misalignment between the discussion of YOLO and the classification task at hand. We appreciate the opportunity to clarify our approach. While the task in our study is indeed classification, we initially explored the YOLO framework, particularly Darknet-19, for its potential in extracting meaningful features in a way that benefits subsequent classification tasks. We initially considered YOLO's object detection capabilities due to its efficiency in feature extraction, which can be applied to classify regions of interest (such as diseased portions of leaves) in images.

**Comment#9. The section 2.2 CNN is unnecessarily lengthy and does not discuss the particular CNN used in this work in detail.**

Response: Thank you for your helpful comment. We agree that Section 2.2 on CNNs was unnecessarily lengthy and did not provide a focused discussion on the specific CNN used in our work. In response to your suggestion, we have revised Section 2.2 to make it more concise and directly relevant to the CNN model employed in this study.

**Comment#10. Feature extraction is automatic in CNN, why separate section is there in the manuscript? Why ResNet, Mobile-net and DarkNet are not used as end-to-end classification model?**

Response: Thank you for your valuable comment. We appreciate your feedback regarding the need for a separate feature extraction section and the use of end-to-end classification models like ResNet, MobileNet, and DarkNet.

In response to your suggestion, we have removed the separate subsection on feature extraction from the revised manuscript. We agree that feature extraction is inherently part of the CNN process, and its distinction as a separate step may have caused confusion. The manuscript now focuses on the CNN architecture used for classification and provides a clear explanation of how MobileNetV2 was employed to extract features as part of the broader classification framework.

Regarding the use of end-to-end models like ResNet, MobileNet, and DarkNet, we opted for a hybrid approach where MobileNetV2 was used for feature extraction followed by traditional machine learning classifiers.

The selection of pre-trained networks, namely DarkNet19, MobileNetV2, and ResNet18, was driven by their well-documented efficacy and suitability for our specific research objectives. These architectures stand out as widely recognized and extensively utilized models in the realm of lightweight deep learning for image classification.

DarkNet19, defined by its depth and effectiveness, is capable of handling exceptionally deep neural networks with over 19 layers. Its architecture, which includes global kernels and channel-doubling post-pooling layers, enables rapid training and validation. Notably, DarkNet19's design addresses the vanishing gradient problem using solutions such as identity mapping, which ensures robust training and allows for effective feature extraction for classification tasks.

MobileNetV2, known for its lightweight design and high performance, was elected because of its exceptional balance of computational economy and classification accuracy. Its simplified architecture enables simple training with little increases in training error percentage, making it an excellent alternative for resource-constrained environments. Furthermore, MobileNetV2's use of identity mapping techniques improves gradient stability, increasing the model's ability to learn complicated features from input photos effectively.

ResNet18, an established architecture stated for its depth and simplicity, contributes to our research goals by striking a compromise between model complexity and performance. Its very shallow structure, with 18 layers, allows for rapid feature extraction while maintaining excellent classification accuracy. ResNet18's skip connections allow for effective gradient propagation, overcoming issues like the vanishing gradient problem and supporting sustained and resilient training.

**Comment#11. Where are the pre-trained models for darknet19 trained?**

Response: Thank you for your insightful comment. Regarding the pre-trained model for Darknet-19, we would like to clarify that we utilized transfer learning in this study.

**Comment#12. can table 1 be better represented?**

Response: Thank you for your suggestion regarding Table 1. We agree that the presentation of the hyperparameters could be improved for better clarity and readability. In the revised manuscript, we have enhanced the structure of Table 1 to provide a clearer representation of the details for the second and third experiments.

**Comment#13. why three ML model SVM, KNN and ensemble are chosen? Why RF or MLP are not selected? Again, what is the configuration of ensemble model? How many base learners are involved there?**

Response: SVM was selected for its effectiveness in high-dimensional feature spaces, which is particularly useful for the complex feature sets extracted from the rice leaf images. KNN was chosen for its simplicity and effectiveness in classification tasks where the decision boundary is non-linear. The ensemble method was chosen for its ability to combine the strengths of multiple classifiers and improve the overall accuracy and robustness of the model.

We employed a majority voting strategy, where each base learner casts a vote for the class, and the class with the majority of votes is selected as the final prediction. This method combines the strengths of both SVM and KNN by leveraging their complementary decision-making processes.

The ensemble approach improves generalization by reducing the likelihood of overfitting to any single model's weaknesses, leading to a more robust and reliable classifier.

**Comment#14. How feature from different CNN are merged? is this simple concatenation or addition or any other technique?**

Response: In our approach, simple concatenation is used to merge the features extracted from the different deep learning models. After obtaining the feature vectors from each model, we concatenate them into a single, unified feature vector that serves as input for the subsequent machine learning classifiers. This technique allows us to combine the strengths of different models and extract complementary features, which can enhance the overall performance of the classification system.

**Comment#15. More experiments are needed with pre-trained networks such as NASNetMObile, EfficientNet, VGG, ConvNetxT, Xception, Inception and so on to see the performance and benchmarking.**

Response: Thank you for your valuable suggestion. We appreciate your input regarding the use of additional pre-trained networks such as NASNetMobile, EfficientNet, VGG, ConvNetX, Xception, Inception, and others. We agree that incorporating these models would provide a comprehensive comparison and benchmarking for the performance of rice disease detection.

While this paper focuses on the use of MobileNetV2, darknet19, resnet and a hybrid machine learning approach, we acknowledge the potential benefits of evaluating other advanced pre-trained networks for this task. We plan to conduct further experiments involving the suggested models in future work to compare their performance and evaluate their effectiveness in this domain.

**Comment#16. The heatmap of each CNN for feature extraction should be visualized with GradCAM to see is there any difference in the features coming from each CNN?**

Response: Thank you for your insightful suggestion regarding the visualization of features extracted from each CNN using GradCAM. We agree that visualizing the feature maps through GradCAM could provide valuable insights into how each CNN model focuses on different parts of the rice leaf images and help compare the effectiveness of each model in terms of learned features. While this analysis was not included in the current study, we believe it would be an important addition to assess the interpretability of the models and their decision-making processes. We plan to incorporate GradCAM visualizations in future work to visually analyze the areas of the image that each CNN model focuses on during feature extraction. This will help us understand if there are any significant differences in the learned features from each CNN and contribute to a more comprehensive evaluation of the models.

**Comment#17. The conclusion about CNN and ML for automated diseases detection is not supported by the results. Please take care of this.**

Response: Thank you for your feedback regarding the conclusion. We understand the importance of ensuring that the conclusion is fully supported by the results presented in the manuscript. In the revised conclusion, we have carefully aligned our discussion with the results from our experiments to ensure a more accurate reflection of the findings. We have emphasized the effectiveness of the proposed hybrid model in detecting rice diseases based on the achieved classification accuracy (98.6%), specificity (98.85%), and sensitivity (97.25%). These results demonstrate the model’s potential to improve disease detection and assist in timely decision-making, ultimately contributing to improved crop productivity. We have revised the conclusion to more clearly state how the model's performance translates into practical benefits for farmers. Specifically, we highlight that the proposed system automates the disease detection process, making it quicker, more reliable, and cost-effective for farmers. The inclusion of specific numerical results provides more concrete support for the claims made in the conclusion. Furthermore, we have adjusted the conclusion to better emphasize the limitations of current image processing techniques and the advantages of our proposed approach, particularly in terms of its efficiency and scalability.

**Comment#18. The state of the art comparison in the manuscript is very poor. Author are suggested to compare their proposal with existing methods such as**

**1) https://www.sciencedirect.com/science/article/pii/S221431732400026X**

**2)** [**https://www.sciencedirect.com/science/article/pii/S0013935121005697**](https://www.sciencedirect.com/science/article/pii/S0013935121005697)**.**

Response: Thank you for your valuable feedback regarding the state-of-the-art comparison. We appreciate your suggestion to compare our proposed approach with existing methods. In response to your comment, we have reviewed the suggested articles and added a comparative table to the revised manuscript.

**…………………………………………………………………………………………………..**

**Reviewer#2: The authors develop a hybrid model for rice disease prediction,the problem is important and interesting.**

Mojor comments:

**Comment#1: The authors didnot compare the proposed model with SOTA model.**

Response: Thank you for your valuable feedback regarding the state-of-the-art comparison. We appreciate your suggestion to compare our proposed approach with existing methods. In response to your comment, we have reviewed the suggested articles and added a comparative table to the revised manuscript.

**Comment#2. The authors should read more related papers on rice disease prediction and review them.**

Response: Thank you for your suggestion. We agree that a thorough review of recent studies on rice disease prediction is essential to enhance the context and depth of our manuscript. In response to your comment, we have reviewed and incorporated additional relevant papers on rice disease prediction, particularly those that focus on recent advancements in image processing, deep learning, and machine learning techniques. These articles have been added in the revised manuscript to provide a more comprehensive overview of the current state of the field.

**Comment#3. The authors should test more dataset to verify the performance of the proposed model.**

Response: Thank you for your valuable suggestion. We agree that testing the proposed model on additional datasets would provide a more comprehensive evaluation of its performance and generalizability. While the current study utilizes a specific dataset, we recognize the importance of validating the model's effectiveness across a broader range of data.

In future work, we plan to test the proposed approach on multiple datasets from different geographical regions and varying crop conditions. This will allow us to assess the robustness of the model and its applicability to real-world scenarios, ensuring that it performs well across diverse rice plant varieties and environmental conditions.

Minor comments:

**Comment#1. F-1 score is different from F1 score, the symbol should keep consistent.**

Response: Thank you for pointing out the inconsistency in the notation. We have corrected the symbol to ensure consistency throughout the manuscript. The term "F1 score" is now used uniformly in all instances where this metric is mentioned.
