# Supplementary material for: Multi-model machine learning for automated identification of rice diseases using leaf image data
Source: PLoS One. 2025 Sep 16;20(9):e0307461. doi: 10.1371/journal.pone.0307461 (PMC12440219; doi:10.1371/journal.pone.0307461)
Supplement: Attachment — Submitted filename: RTR_for_Rice_leaf_auresp_2.docx [file pone.0307461.s002.docx]

**RESPONSES TO REVIEWERS’ COMMENTS AND SUGGESTIONS**

**Journal: PLOS ONE
Manuscript Number: PONE-D-24-27739R1
Article Type: Full-Length Article
Title: Multi-Model Machine Learning for Automated Identification of Rice Diseases Using Leaf Image Data**

We thank reviewers, academic editor, and the editor for their time, constructive comments, and useful suggestions. We have addressed the comments of the reviewers to the best of our ability. Our responses to the reviewers’ comments (bold letter) are in blue and in red are the addition, deletion, or modification in the revised manuscript.

**Reviewer #3: The manuscript proposes a new machine learning framework for classification of rice disease leaves. The framework is arguably unexplored, but my main concerns come from 1) the lack of clarity in the methodology carried out in the study and 2) the lack of baseline class included in the experiment. In the past, it is acceptable to propose classification model for “disease” classes only. However, recently, for models/frameworks to be comparable and generalizable, healthy class of the dataset should also be included to establish baseline for performance as each framework may use different sets of train and test datasets. Please see comments by section below.**

**Comment#1: Method does not reflect the final proposed framework. The abstract explains that the author uses MobileNetV2 architecture, but the final proposed framework presents that merged features from three CNN models gives the best performance.**

Response: Thank you for the comment. In this study, we extracted deep features from rice leaf images using pre-trained CNN models—MobileNetV2, DarkNet19, and ResNet18—and classified them using machine learning classifiers. As suggested, we have revised the abstract to accurately reflect the proposed multi-model framework used in the final analysis.

**Comment#2: “Medium” neural network is not a standard terminology. It has no meaning for the abstract if no context is given.**

Response: Thank you for pointing this out. The term “medium” referred to the medium Gaussian kernel of the SVM classifier, not a neural network. We have revised the abstract to clarify this and avoid any confusion by explicitly referring to the SVM with a medium Gaussian kernel.

**Comment#3: “world-class” is used without justification in comparison to other established performance in the field. For example, in this case, the author uses Kaggle dataset, is this framework performs better than all previous Kaggle competition on this same dataset?**

Response: As suggested, we have removed the term “world-class” from the manuscript. The revised version now reports the performance metrics objectively: using a medium Gaussian kernel of the SVM classifier, the proposed system achieved a classification accuracy of 98.61%, specificity of 98.85%, and sensitivity of 97.25%.

**Comment#4: Conclusion for the abstract should not include future work. Abstract should summarize the main argument and findings of the study.**

Response: We have removed the mention of future work from the abstract to ensure it focuses solely on the main findings of the study.

**Comment#5: Conclusion argues that the technique provides solution for early diagnosis, but no information given in the manuscript with regards to the age of the leaves of the images used for training/test.**

Response: We acknowledge that the dataset used (Kaggle – Rice Leaf Disease Dataset) does not provide information regarding the age of the leaves. Therefore, we have revised the conclusion to avoid referring to “early diagnosis” and instead emphasize accurate classification of rice leaf diseases based on the available image data.

**Comment#6: Arguments are repeated in the section. For example, “As a result, there is a critical need for disease diagnosis and management…” and “To classify and diagnose different diseases at an early stage and with higher accuracy, …” support the same argument. I suggest reorganization of the section for a paragraph to convey one argument.**

Response: We have restructured the section to eliminate redundancy and ensure that each paragraph conveys a single clear argument. The repetitive statements have been consolidated, and the paragraph has been reorganized for better flow and clarity.

**Comment#7: Paragraph 2 is very long and hard to understand as it argues multiple points. Some sentences are not clear such as “farmers in rural areas to have to travel great distances to discover crop diseases.” Does this mean farmers bring leaves to classify diseases or they travel around in a large field until they find crops that have diseases?**

Response: Thank you for the helpful feedback. We have revised Paragraph 2 to improve clarity and readability by breaking it into shorter, more focused sentences. Specifically, the sentence about farmers traveling great distances has been clarified to explain that it refers to the need for farmers to visit multiple locations in rural areas to identify crop diseases, which can be time-consuming and inefficient. We have updated this to make the point clearer.

**Comment#8: Previous work section explains multiple related studies, but do not specify performance of those studies, preferably in exact numbers, nor the characteristics of the datasets used. The two components are important for comparing and contract with the study.**

Response: We have revised the previous work section to include the performance metrics (accuracy, specificity, sensitivity, etc.) of the studies cited, along with details on the datasets used (e.g., dataset size, image types, and any preprocessing applied). This will allow for a clearer comparison between the existing literature and our study.

**Comment#9: Previous work section should be broken down into multiple paragraphs. One paragraph presents one component that is influencing the design of the study. The current format of the section contains multiple studies, one after another, and is not clear how the referenced studies relate to the manuscript.**

Response: We have reorganized the previous work section into multiple paragraphs, each focusing on a specific component influencing the design of our study. This structure now clearly highlights how each referenced study relates to the objectives and methodology of our research.

**Comment#10: Grammatical issues such as “like as”, “In the domain of diseases.., In 18 confirmed..”.**

Response: The manuscript has been proofread by a native English speaker, and all typos and grammatical errors have been corrected in the revised paper.

**Comment#11: Some studies mentioned in the Previous work section seem unrelated to the manuscript. Such as studies involved UAVs without explaining the characteristic of the images compared to the rice disease leaves.**

Response: As suggested, we have removed the unrelated studies from the previous work section, ensuring that only relevant research related to rice disease leaf images is discussed.

**Comment#12: The final part of the Previous work section details the key contributions of the study. I would suggest move this part to Discussion and instead details the study and how the study is different from previous works explained in the section.**

Response: We have moved the discussion of the key contributions of our study to the Discussion section, as recommended. The Previous work section now focuses solely on detailing related studies and how our approach differs from them.

**Comment#13: In the datasets section explains how the dataset is gathered without detailing exactly how the dataset is curated. For example, “rigorous quality control measures were implemented” is mentioned, but what are the QC controls – is it image size being standardized? Exact details of the dataset should also be clearly explained. Standard property such as image size and format should be specified.**

Response: In the revised manuscript, we have expanded the dataset section to include specific details on the curation process and the quality control measures implemented. This includes the standardization of image dimensions (e.g., resized to 224×224 pixels), removal of blurry or low-quality images, and ensuring uniform lighting and background conditions. We have also specified the image format used (JPEG) and clarified the distribution of images across classes. These details are now provided to improve the transparency and reproducibility of the dataset preparation.

**Comment#14: The method of how features from different CNN are merged is not explained. This should be shown in mathematical representation as CNN features are in matrices.**

Response: In the revised manuscript, we have elaborated on the method used for merging the features extracted from the different CNN models—MobileNetV2, DarkNet19, and ResNet18.

**Comment#15: Three classes are being classified, but to establish baseline a class of healthy leaves should also be included.**

Response: In the revised version of the manuscript, we have included a separate class of healthy rice leaves in our dataset to enable comparative classification. Specifically, 1280 images of healthy rice leaves were added to the dataset, ensuring that the model learns to distinguish between diseased and non-diseased conditions. Consequently, the classification task now encompasses four categories: bacterial blight, leaf blast, brown spot, and healthy. All experimental results, performance metrics, and confusion matrices have been updated accordingly in the manuscript to reflect this important inclusion.

**Comment#16: The excerpt “when the input image quality satisfies the predetermined standards” – please detail the image quality requirements/predetermined standards in exact quantitative metrics with numbers. These are important steps for the study to be useful for other readers in the field.**

Response: In the revised manuscript, we have elaborated on the predetermined image quality standards used during the preprocessing phase. Specifically, images were required to meet the following quantitative criteria: (i) minimum resolution of 256 × 256 pixels to ensure sufficient feature representation; (ii) file format restricted to JPEG or PNG with a compression ratio not exceeding 10:1 to avoid loss of visual features; (iii) brightness values within the normalized intensity range of 0.3 to 0.8 to prevent overly dark or bright images; (iv) absence of motion blur or shadow occlusions as determined through Laplacian variance thresholding (≥100 for sharpness). Images failing to meet these standards

**Comment#17: For the CNN section, the paragraph explains the reasons why the author chose each framework (some models mentioned also were not used in the study), but do not explain the actual method of the CNN models used in the study. The reasons why the author chose the framework should be in previous study section and not in the materials and methods section. Materials and Methods section should detail the method used in the study. As there are three CNN models in the study, I suggest separate each model into each subsection for clarity.**

Response: We thank the reviewer for this valuable suggestion. In the revised manuscript, we have restructured the *Materials and Methods* section to enhance clarity and focus on the technical implementation. Specifically, we have removed the justification for model selection from this section and relocated it to the *Previous Work* section, where relevant prior studies are discussed. Furthermore, to improve readability and organization, we have divided the explanation of the three CNN models—MobileNetV2, DarkNet19, and ResNet18—into separate subsections. Each subsection now

**Comment#18: Please clearly explain ensemble methods employed in the study, specifically which base models are used. The methods currently listed, namely Bagging, Subspace, RUSBoosted, are techniques of ensembling, not base models. If the author ensembled from the other classifier namely SVM and KNN, then do specify that clearly.**

Response: In the revised manuscript, we have clarified the ensemble methods used in our study by explicitly specifying the base learners involved in each ensemble technique. For Bagging, Subspace, and RUSBoosted ensemble methods, the base classifiers employed were decision trees. We have also confirmed that SVM and KNN were used as standalone classifiers for performance comparison, but they were not used as base learners in the ensemble frameworks. This clarification has been incorporated into the *Materials and Methods* section to ensure methodological transparency and reproducibility.

**Comment#19: This section contains a part of methodology and a part of results. I would suggest separate a Result/Findings section for clarity.**

Response: Based on the recommendation, we have revised the manuscript by separating the content related to methodology and results into distinct sections.

**Comment#20: data augmentation techniques should explain the final number of the images at the end of the process. Each technique should be detailed. For example, a range of horizontal-vertical shear is mentioned, but which numbers were used exactly?**

Response: In the revised manuscript, we have clearly detailed each data augmentation technique applied, including the exact parameters used. Specifically, we employed horizontal flip (probability = 0.5), vertical flip (probability = 0.5), random rotation (angles between -5° to +5°), zoom range (0.8 to 1.2), width and height shift (range = 0.2), and shear transformation (shear range = 0.2 for both horizontal and vertical directions). After applying these augmentation techniques, the dataset size was increased from the original 3,696 images to a final count of 11,088 images, ensuring a balanced representation across all three disease classes. These details have been incorporated into the Data Augmentation subsection for clarity and reproducibility.

**Comment#21: 10-fold cross validation explained in the section is not the normal practice of cross-validation technique. From the explanation, the author divides the extracted features into ten equal parts. This might be a language issue, but my interpretation here implies some “features” from the CNN step are missing from the training/testing/validation step. Cross-validation technique divides the whole dataset into train/test/validation, not features.**

Response: In the revised manuscript, we have clarified that the 10-fold cross-validation technique was applied to the entire dataset, not only the extracted features. Specifically, the dataset was randomly divided into 10 equal subsets (or folds), and each fold was used as the validation set once, while the remaining nine folds were used for training. This process was repeated for all 10 folds, ensuring that all data points were used for both training and validation.

**Comment#22: Table 1 and Table 2 list other ensemble methods that were not mentioned in the manuscript before. Which were experimented exactly? The lines were not aligned with results. It is hard to link results to the ensemble method.**

Response: We acknowledge that the ensemble methods listed in Table 1 and Table 2 were not adequately explained in the manuscript before the tables, leading to confusion in linking them with the results. In the revised manuscript, we have clarified which ensemble methods were actually experimented with. Specifically, we only used the ensemble methods that were explicitly mentioned in the methodology section, such as Bagging, Subspace, and RUSBoosted. The other methods listed in the tables were inadvertently included and have been removed.

**Comment#23: The author should specify why they chose accuracy as the main metrics to judge the performance. I suggest Table 3 to also lists results of other metrics for all models that were experimented as well.**

Response: Thank you for the suggestion. We chose accuracy as the primary metric because it provides a straightforward and effective measure of overall classification performance, particularly for the rice plant disease classification task, where the goal is to classify each image correctly into one of the disease categories. Accuracy has been widely used in similar studies, making it a standard metric for comparison.

**Comment#24: Figure 5 is not the correct use of pie chart. The metrics are different and are not appropriate to be comparable purely on the same set of models.**

Response: Thank you for your valuable feedback. Based on your suggestion, we have removed Figure 5 from the revised paper.

**Comment#25: I suggest the author to add confusion metrics to the results. It will clearly represent the performance of the framework by class and also to compare with other frameworks, not only just the best one.**

Response: In response, we have added the confusion matrix to the results section of the revised paper. The inclusion of the confusion matrix allows for a clearer representation of the framework's performance on a per-class basis and provides a more comprehensive comparison with other frameworks, rather than focusing solely on the best-performing model.

**Comment#26: The first paragraph of Discussion should summarize the result of the study and follow by compare and contrast of the performance with previous studies. Exact numbers are encouraged. Table 4 which lists the performance of other studies is a good base, but the table lacks detail of the dataset. Upon checking, the second and the third studies are not rice leaves. In the case that there are no comparable studies, explanation should be provided how the other plants are comparable to rice leaves.**

Response: In response to your suggestion, we have revised the first paragraph of the Discussion section to first summarize the results of our study, followed by a detailed comparison of the performance with previous studies. We have included exact numbers to provide a clearer and more meaningful comparison.

………………………………………………………………………………………………….

**Reviewer #4: The authors have addressed all reviewer comments to the satisfaction of the reviewers, as evidenced by their revised manuscript.**

Response: Thank you for your valuable feedback and recommendation.
