# Supplementary material for: Multi-model machine learning for automated identification of rice diseases using leaf image data
Source: PLoS One. 2025 Sep 16;20(9):e0307461. doi: 10.1371/journal.pone.0307461 (PMC12440219; doi:10.1371/journal.pone.0307461)
Supplement: Attachment — Submitted filename: RTR_for_Rice leaf - Copy.docx [file pone.0307461.s003.docx]

**RESPONSES TO REVIEWERS’ COMMENTS AND SUGGESTIONS**

**Journal: PLOS ONE
Manuscript Number: PONE-D-24-27739R3
Article Type: Full-Length Article
Title: Multi-Model Machine Learning for Automated Identification of Rice Diseases Using Leaf Image Data**

We thank reviewers, academic editor, and the editor for their time, constructive comments, and useful suggestions. We have addressed the comments of the reviewers to the best of our ability. Our responses to the reviewers’ comments (bold letter) are in blue and in red are the addition, deletion, or modification in the revised manuscript.

****No any comments from the reviewers.**
